# *Candida auris*: Epidemiology, biology, antifungal resistance, and virulence

**DOI:** 10.1371/journal.ppat.1008921

**Published:** 2020-10-22

**Authors:** Han Du, Jian Bing, Tianren Hu, Craig L. Ennis, Clarissa J. Nobile, Guanghua Huang

**Affiliations:** 1 Department of Infectious Diseases, Huashan Hospital and State Key Laboratory of Genetic Engineering, School of Life Sciences, Fudan University, Shanghai, China; 2 Institutes of Biomedical Sciences, Fudan University, Shanghai, China; 3 Quantitative and Systems Biology Graduate Program, University of California, Merced, Merced, United States of America; 4 Department of Molecular and Cell Biology, University of California, Merced, Merced, United States of America; Rutgers University, UNITED STATES

## Abstract

First described in 2009 in Japan, the emerging multidrug-resistant fungal pathogen *Candida auris* is becoming a worldwide public health threat that has been attracting considerable attention due to its rapid and widespread emergence over the past decade. The reasons behind the recent emergence of this fungus remain a mystery to date. Genetic analyses indicate that this fungal pathogen emerged simultaneously in several different continents, where 5 genetically distinct clades of *C*. *auris* were isolated from distinct geographical locations. Although *C*. *auris* belongs to the CTG clade (its constituent species translate the CTG codon as serine instead of leucine, as in the standard code), *C*. *auris* is a haploid fungal species that is more closely related to the haploid and often multidrug-resistant species *Candida haemulonii* and *Candida lusitaniae* and is distantly related to the diploid and clinically common fungal pathogens *Candida albicans* and *Candida tropicalis*. Infections and outbreaks caused by *C*. *auris* in hospitals settings have been rising over the past several years. Difficulty in its identification, multidrug resistance properties, evolution of virulence factors, associated high mortality rates in patients, and long-term survival on surfaces in the environment make *C*. *auris* particularly problematic in clinical settings. Here, we review progress made over the past decade on the biological and clinical aspects of *C*. *auris*. Future efforts should be directed toward understanding the mechanistic details of its biology, epidemiology, antifungal resistance, and pathogenesis with a goal of developing novel tools and methods for the prevention, diagnosis, and treatment of *C*. *auris* infections.

## Introduction

Fungal infections are increasingly recognized as a worldwide threat to human health. About 1.7 billion people worldwide suffer from a fungal infection, most of which are superficial infections of the skin and mucosa (reviewed by [1]). *Candida* species are the predominant cause of nosocomial fungal infections and are the fourth leading cause of all hospital-acquired infections [2]. Annually, there are approximately 400,000 bloodstream infections caused by *Candida* species globally, with mortality rates exceeding 40% [1]. The most frequently encountered *Candida* species is *Candida albicans*; however, the incidence of non-*albicans* species, such as *Candida tropicalis*, *Candida parapsilosis*, and *Candida glabrata*, has increased over recent decades due to the long-term use and limited options of antifungal drugs [3,4].

**Fig 1:**
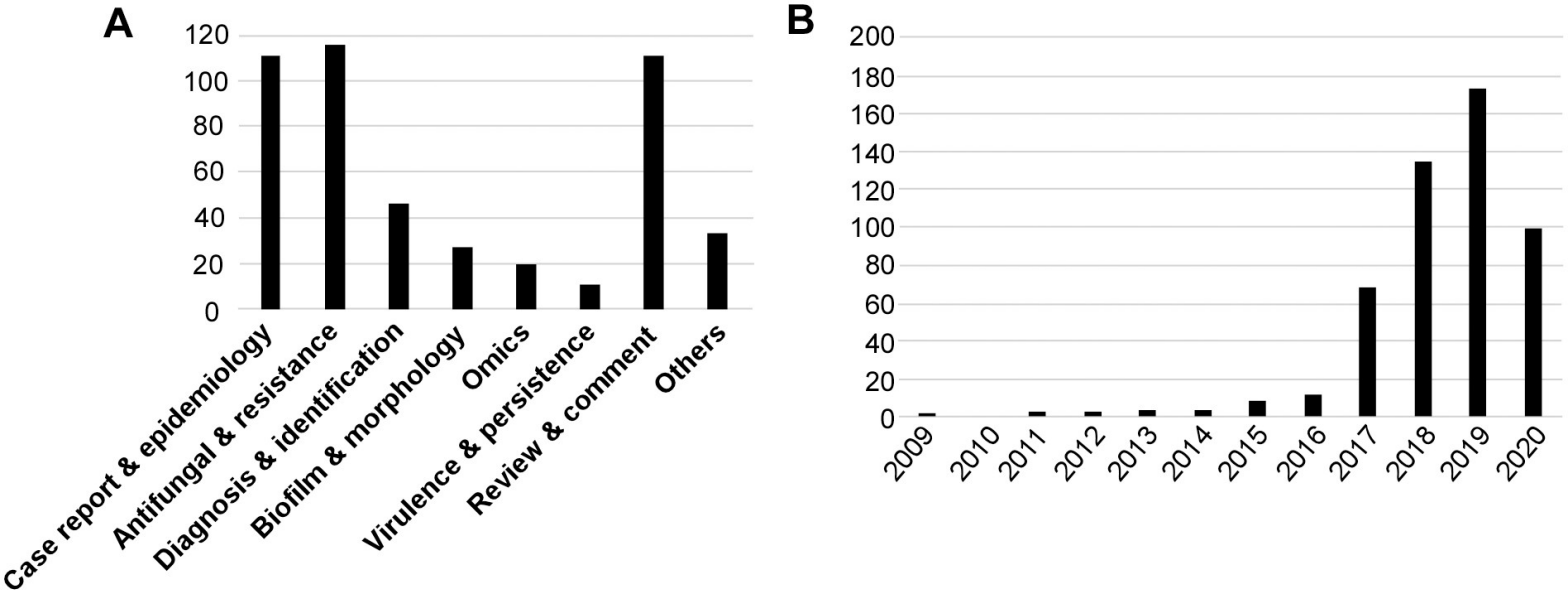
A review of published literature on *C*. *auris* between January 2009 and June 2020. (A) Literature published on topics pertaining to *C*. *auris* since its first identification. (B) Number of published articles in each year. Data for the months January to June were collected for the year 2020. A search of published papers between January 2009 and June 2020 was performed using PubMed and Web of Science databases. The terms “*Candida auris*” or “*C*. *auris*” were used as keywords for database searches. Non-related studies and studies not published in English were excluded from this analysis.

**Fig 2:**
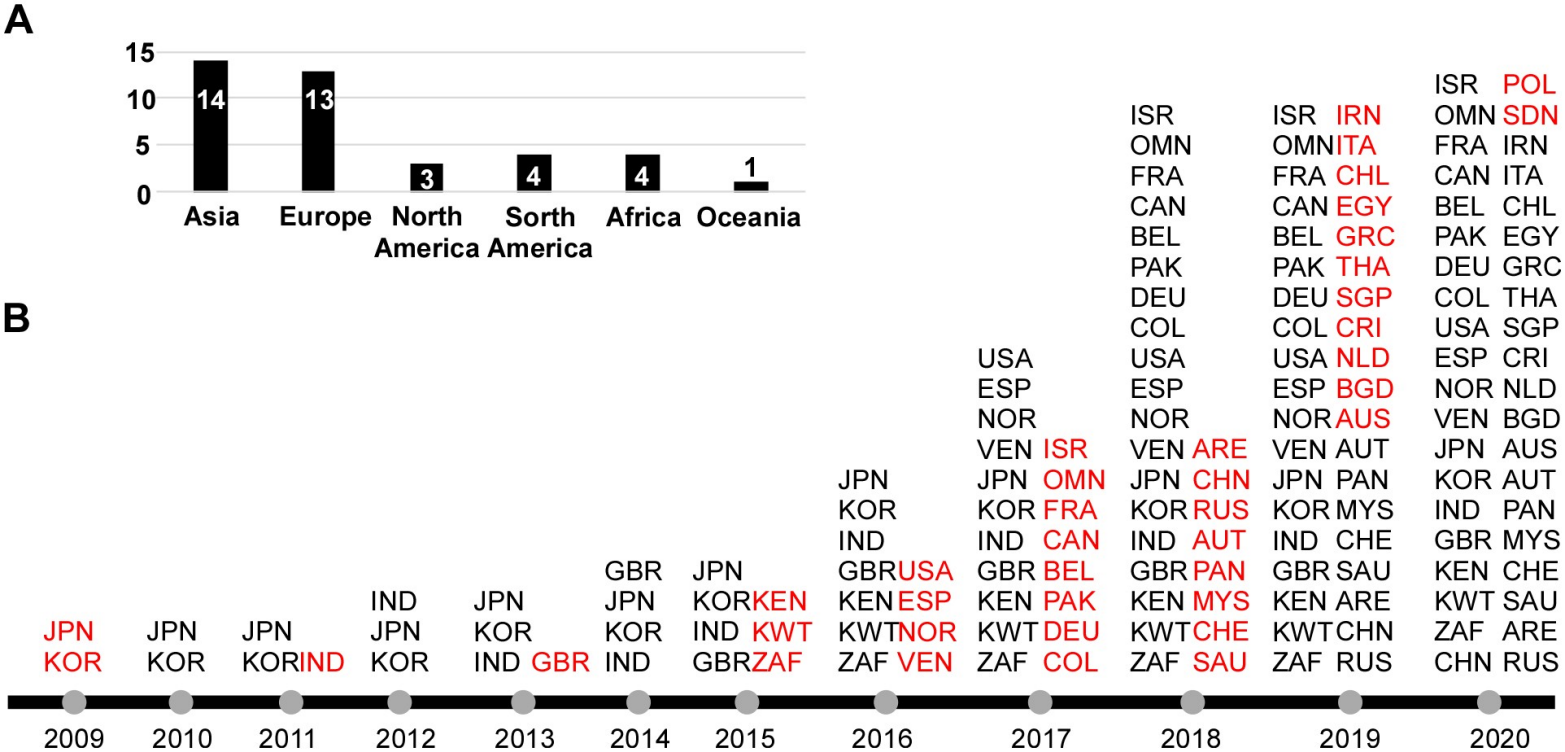
Countries with reported cases of *C*. *auris* infection or colonization from January 2009 to June 2020. (A) Number of countries belonging to each continent that have reported infection or colonization with *C*. *auris*. (B) Countries with reported cases from January 2009 to June 2020. The first reported case from each country is denoted in red text. ARE, United Arab Emirates; AUS, Australia; AUT, Austria; BEL, Belgium; BGD, Bangladesh; CAN, Canada; CHE, Switzerland; CHL, Chile; CHN, China; COL, Colombia; CRI, Costa Rica; DEU, Germany; EGY, Egypt; ESP, Spain; FRA, France; GBR, United Kingdom; GRC, Greece; IND, India; IRN, Iran; ISR, Israel; ITA, Italy; JPN, Japan; KEN, Kenya; KOR, Korea (South); KWT, Kuwait; MYS, Malaysia; NLD, the Netherlands; NOR, Norway; OMN, Oman; PAK, Pakistan; PAN, Panama; POL, Poland; RUS, Russia; SAU, Saudi Arabia; SDN, Sudan; SGP, Singapore; THA, Thailand; USA, United States of America; VEN, Venezuela; ZAF, South Africa.

*Candida auris* is a newly emerged member of the *Candida*/*Clavispora* clade, first isolated in Japan in 2009 from the ear discharge of a female patient [5]. In the past decade, infections caused by *C*. *auris* have become a global threat due to its rapid emergence worldwide and multidrug resistance properties. In 2016, the Centers for Disease Control and Prevention (CDC) released a clinical alert to healthcare facilities warning of the international emergence of *C*. *auris* infections with high mortality rates, and in 2017 provided an update on *C*. *auris* spread throughout the United States of America with disinfection information and treatment recommendations. From its discovery in 2009 until June 2020, *C*. *auris* has attracted considerable attention from both clinical and basic science research fields. Indeed, within that time frame, nearly 500 scientific articles have been published related to *C*. *auris* based on PubMed and Web of Science databases (Fig 1). As of this year, based on published literature and data from the CDC (https://www.cdc.gov/), *C*. *auris* has been isolated in over 40 countries across 6 continents (Fig 2). It has also led to several recent outbreaks in hospitals across the globe [6–9]. Of further concern is the fact that most clinical isolates are resistant to 1 or more classes of the antifungal drugs typically used to treat *Candida* infections [10,11]. Taken together, its multidrug resistance, rapid global emergence, and high mortality rates make *C*. *auris* a particularly problematic pathogen that has garnered considerable attention from the public, medical community, and basic research scientists. Here, we review the identification, epidemiology, clinical manifestations, risk factors, biology, antifungal resistance mechanisms, virulence, genomics and genetics, and origins of *C*. *auris*.

## Identification

*C*. *auris* was first isolated from the ear canal of a Japanese patient and thus named “auris” [5]. A retrospective study revealed that the earliest isolate of *C*. *auris* dates back to 1996, where it was initially misidentified in South Korea as *Candida haemulonii* [12]. Cases of *C*. *auris* infections, however, were rare before 2009, suggesting that this fungus is a newly evolved pathogen.

**Fig 3:**
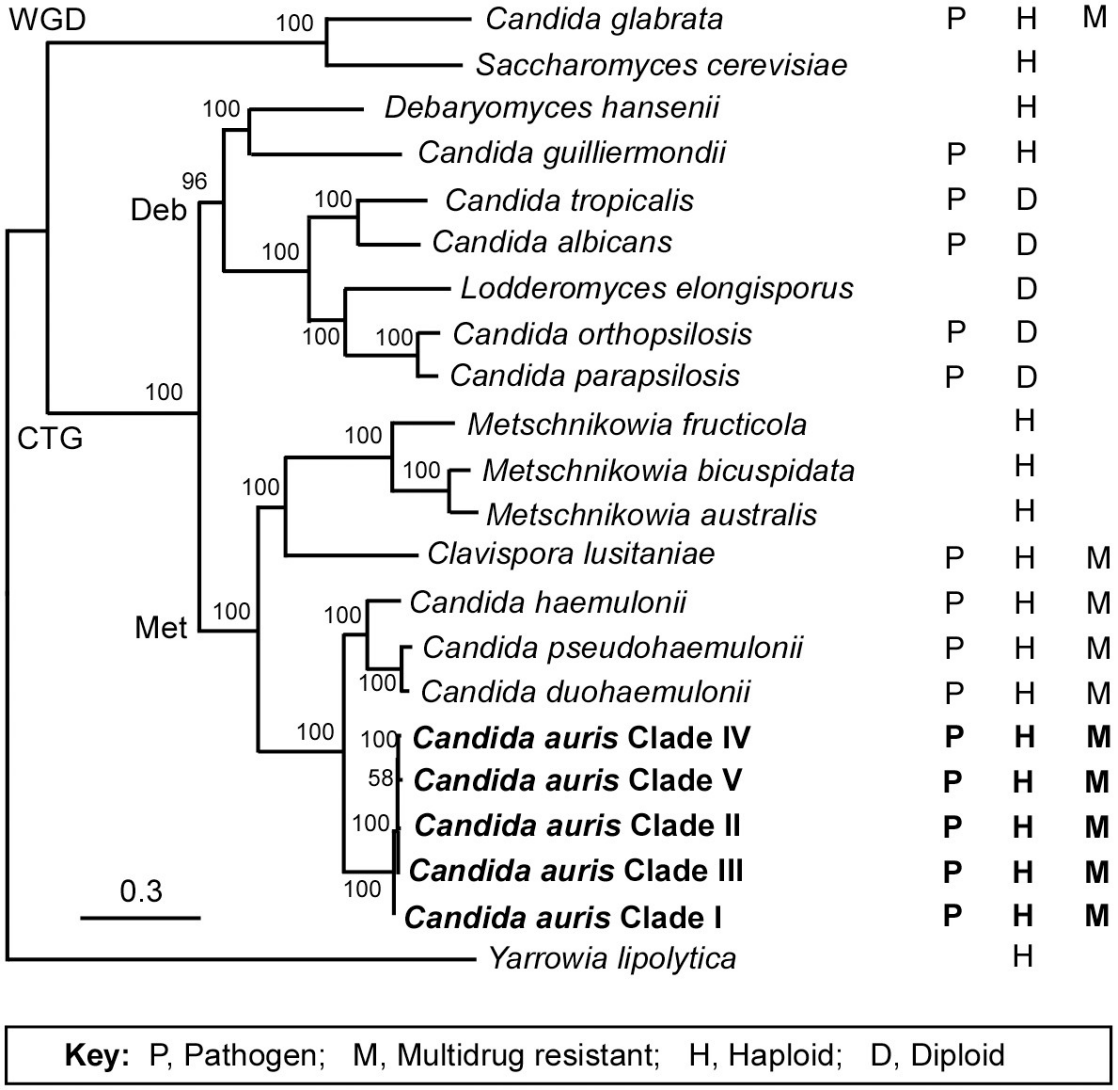
Maximum-likelihood phylogeny of the CTG and WGD clade species. The phylogenic tree was generated using the program RAxML v7.3.2 using 50 protein sequences aligned with Mafft-homologs. The GTR model, gamma distribution, and 1,000 bootstraps were used to construct the phylogenetic relationships. Pathogenic characteristics (P), ploidy (H or D), and multidrug resistance (M) for each species are also shown. CTG, the CTG clade; Deb, Debaryomycetaceae; GTR, generalized time reversible; Met, Metschnikowiaceae; WGD, the Whole Genomic Duplication clade.

Analysis of rDNA sequences of the 28S D1/D2 and 18S internal transcribed spacer (ITS) regions and 50 protein sequences indicates that *C*. *auris* belongs to the Metschnikowiaceae family within the *Candida/Clavispora* clade (Fig 3) [5,13,14]. *C*. *auris*, like other species of the *Candida/Clavispora* clade, such as *C*. *albicans*, *C*. *tropicalis*, *C*. *haemulonii*, and *Candida lusitaniae*, is a member of the CTG clade. Species within this clade translate the CTG codon as serine rather than leucine [15].

*C*. *auris* can be easily misidentified as *C*. *haemolonii* or other yeast species using conventional phenotypic and biochemical methods [16,17]. The growth of *C*. *auris* on commercial CHROMagar medium (CHROMagar, Paris, France) at temperatures up to 42°C results in white, pink, or dark purple colonies [18–20]. Unlike other *Candida* species, *C*. *auris* grows well at 42°C, and thus this thermal tolerance property is being used to differentiate *C*. *auris* from other *Candida* species [21]. Matrix-assisted laser desorption ionization-time of flight mass spectrometry (MALDI-TOF MS) devices can accurately differentiate *C*. *auris* from other fungal species; however, the accurate identification of *C*. *auris* is dependent on the reference databases included with the MS device [16,22,23]. Polymerase chain reaction (PCR) and molecular techniques are also widely used for *C*. *auris* identification [6,24–27]. Molecular methods based on sequencing of genetic loci, such as the D1/D2 region of the 28S rDNA or the ITS region of rDNA, can accurately detect *C*. *auris* isolates. Future efforts should combine the rapid identification of *C*. *auris* isolates with an assessment of their antifungal drug resistance properties.

### Trends in epidemiology: The rapid global emergence of *C*. *auris*

After the first reports of *C*. *auris* infections in clinical settings, a retrospective study was performed in South Korea that found that the earliest isolates of *C*. *auris* date back to 1996 [17]. These isolates were previously misidentified as *C*. *haemulonii*. Analysis of an international surveillance culture collection of *Candida* isolates (by the SENTRY Antimicrobial Surveillance Program, 15,271 *Candida* isolates, collected between 2004 and 2015) identified 4 *C*. *auris* isolates that were collected in 2009, 2013, 2014, and 2015, indicating that *C*. *auris* appears to be a recently emerged pathogen [10,28]. This idea is further supported by genomic analyses estimating that the most recent common ancestor of *C*. *auris* arose as late as 360 years ago and as early as 38 years ago for different *C*. *auris* subclades. Two studies in India have reported the identification of several new clonal strains of *C*. *auris* as well as amphotericine B- and fluconazole-resistant isolates [29,30]. Subsequently, *C*. *auris*–associated infections have been reported in South Africa, Europe, and America [31–33]. In 2016, the CDC, the European Centre for Disease Prevention and Control (ECDC), and Public Health England released a series of alerts to inform healthcare providers about *C*. *auris* as a new infectious agent. Lockhart and colleagues (2017) published the landmark study reporting the genomic and epidemiological analyses of different genetic populations of *C*. *auris* strains that emerged nearly simultaneously across 3 different continents [10]. *C*. *auris* isolates have since emerged worldwide in at least 40 countries to date (Fig 2).

**Fig 4:**
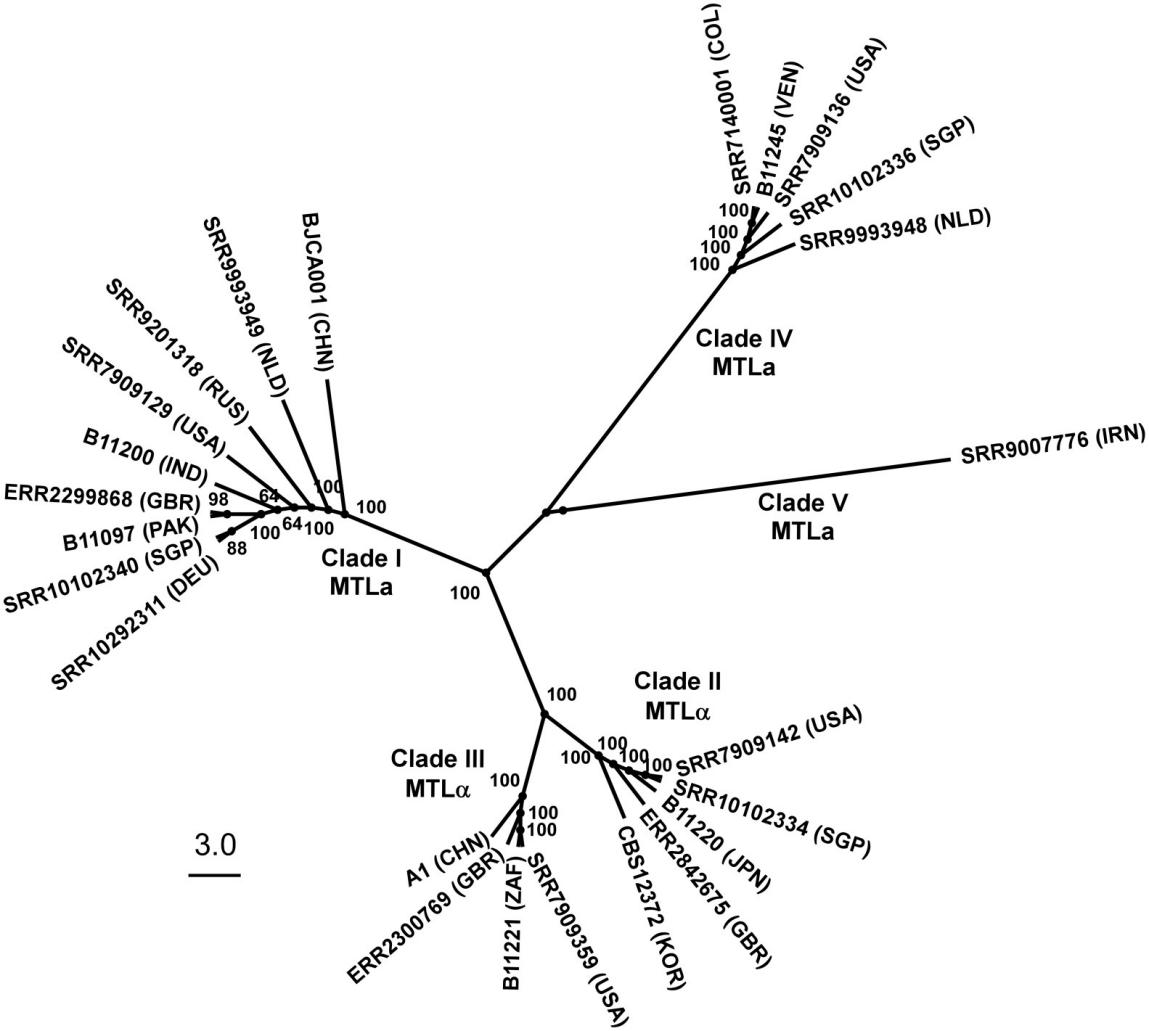
Five clades of *C*. *auris*. The phylogenic tree was generated with the program RAxML v7.3.2 using SNPs. The GTR model, gamma distribution, and 1,000 bootstraps were used to construct the phylogenetic relationships. The MTL are also included for each clade. CHN, China; COL, Colombia; DEU, Germany; GBR, United Kingdom; GTR, generalized time reversible; IND, India; IRN, Iran; JPN, Japan; KOR, Korea (South); MTL, mating type loci; NLD, the Netherlands; PAK, Pakistan; RUS, Russia; SGP, Singapore; SNPs, single-nucleotide polymorphisms; USA, United States of America; VEN, Venezuela describe the country where the strain was first isolated; ZAF, South Africa.

There are 4 major discrete genetic clades of *C*. *auris* based on genetic and genomic information and locations of first isolates: the South Asia Clade (I), the East Asia Clade (II), the South Africa Clade (III), and the South America Clade (IV) (Fig 4). Within each clade, sequencing data indicate that there are very few single-nucleotide polymorphisms (SNPs), typically less than 70 SNPs [10]. Recently, a potential new *C*. *auris* clade (Clade V, Fig 4) has been reported in Iran; interestingly, this clade is separated from the other clades by greater than 200,000 SNPs [34].

### Clinical manifestations

*C*. *auris* has been isolated from multiple infection sites throughout the body and is generally hospital acquired. Clinicians have isolated it from urine, bile, blood, wounds, the nares, the axilla, the skin, and the rectum of infected individuals (reviewed by [16,35]). Unlike *C*. *albicans*, which colonizes the gastrointestinal (GI) and genitourinary tracts of the most healthy individuals, *C*. *auris* is hypothesized to predominantly colonize the skin; however, in rare instances, it has been isolated from the gut, oral, and esophageal mucosa of infected individuals [10]. Consistent with the rarity of isolating *C*. *auris* in the gut, clinical manifestations and in vivo experiments together suggest that *C*. *auris* is incapable of colonizing anaerobic environments like the gut [36]. In terms of the oral mucosa, a recent study found that the salivary antimicrobial peptide histatin 5 has a potent antifungal effect on *C*. *auris* [37]. This peptide may limit the colonization of the *C*. *auris* in the oral mucosa and explain with it is rarely isolated from this area. In clinical settings, *C*. *auris* is most commonly associated with bloodstream infections [22]. One study found that approximately 5% of candidemia cases in intensive care units (ICUs) in India were caused by *C*. *auris* [38]. Invasive infections caused by *C*. *auris* occur more frequent in critically ill patients in ICUs. Similar to other invasive *Candida* infections, invasive *C*. *auris* infections are associated with high global mortality rates ranging from 30% to 60% [10,39,40].

### Risk factors

Risk factors for *C*. *auris* infections are similar to those for other *Candida* species. This is not surprising given that many *Candida* species are opportunistic pathogens and are primarily associated with critically ill and immunocompromised patients. Risk factors for *C*. *auris* infections include elderly age, diabetes mellitus, recent surgery, the presence of an indwelling medical device (e.g., central venous catheter), an immunosuppressed state, the use of hemodialysis, a neutropenic state, chronic renal disease, or the use of broad-spectrum antibiotic and/or antifungal drugs [6,7,23,38,41,42]. In a study that retrospectively analyzed available patient data, it was determined that an increase in *C*. *auris* colonization or infection was associated with diarrhea and the use of the broad-spectrum antibiotic tetracycline as well as the second-generation tetracycline derivatives minocycline and tigecycline [42]. These studies highlight a diverse set of risk factors associated with *C*. *auris* infections.

### Biology

Pathogenic *Candida* species, such as *C*. *albicans*, *C*. *tropicalis*, *C*. *parapsilosis*, and *C*. *auris*, but not *C*. *glabrata*, belong to the CTG clade. Species within this clade translate the CTG codon into serine instead of leucine [15]. Similar to other *Candida* species, *C*. *auris* can form biofilms, undergo filamentation, and phenotypically change between specific cell types [12,32,43–45]. These characteristics may be associated with virulence, antifungal tolerance, and survival in natural and host niches.

#### Adaptation to environmental stresses

There are an estimated 1.5–5.1 million fungal species on Earth ranging from single-celled yeasts to multicellular fungi [46]. Interestingly, most fungi are unable to survive at human physiological temperatures (36.5–37.5°C and up to 40°C during a fever) and are thus unable to colonize humans and cause infections. Strikingly, it has been found that unlike its closely related *Candida* species, *C*. *auris* can grow at high temperatures (>40°C) [41,47,48]. Indeed, a recent study comparing the temperature tolerance of *C*. *auris* to other *Candida* species hypothesized that climate change, specifically global warming, may have contributed to the evolution of *C*. *auris* as a human pathogen and to its ability to grow at high temperatures [21].

Another trait of *C*. *auris* is its ability to tolerate high salt concentrations (>10% NaCl, wt/vol) compared to other *Candida* species [41,48]. Two studies found that *C*. *auris* forms pseudohyphae-like morphologies in response to high salt concentrations, which suggests that this morphological transition may be adaptive under stressful conditions [41,48].

Thermotolerance and osmotolerance are characteristics that may contribute to the persistence and survival of *C*. *auris* on biotic and abiotic surfaces for long periods of time [8,49,50]. Indeed, *C*. *auris* is known to survive on human skin and environmental surfaces for several weeks and can even tolerate being exposed to some commonly used disinfectants. Persistence on surfaces may contribute to the frequently observed intrahospital transmission of *C*. *auris* within healthcare settings. For example, an outbreak of *C*. *auris* at the neurosciences ICU of the Oxford University Hospitals in the United Kingdom was linked to the use of reusable axillary temperature probes [51]. Persistence in harsh environmental conditions is a hallmark feature of *C*. *auris* that distinguishes it from the majority of other human fungal pathogens.

#### Morphological transitions

Morphological plasticity is a common strategy used by microorganisms to rapidly adapt to environmental changes [52,53]. Both bacterial and fungal species can undergo morphological transitions under certain environmental conditions. Pathogenic *Candida* species such as *C*. *albicans* and *C*. *tropicalis* can undergo a number of morphological transitions [54–56]. They can switch between several different cell types spontaneously or in response to environmental cues. Two well-characterized morphological transitions in *C*. *albicans* and *C*. *tropicalis*, for example, are the yeast–hyphal transition and the white-opaque switch. In these species, morphological plasticity plays critical roles in pathogenesis and mating [54–56].

Like other pathogenic *Candida* species, *C*. *auris* also has several morphological phenotypes [12,19,41,43], although the regulatory mechanisms and roles of each morphology in *C*. *auris* are largely unknown. Many isolates of *C*. *auris* exist in the single-cell yeast form. However, a portion of natural *C*. *auris* isolates can form large aggregates of pseudohyphal-like cells, where mother and daughter cells remain attached [32,44]. These aggregates are generally more tolerant to antifungal agents than their non-aggregating counterparts; however, aggregating cells display reduced virulence compared to non-aggregating cells in the *Galleria mellonella* infection model [32]. The formation of these pseudohyphal-like aggregates in *C*. *auris* could be due to a defect in cell division. Consistent with this hypothesis, a recent study demonstrated that induction of DNA damage and perturbation of replication forks by genotoxic stresses promoted pseudohyphal-like formation in *C*. *auris* [57].

#### Colony phenotypic switching

It was recently demonstrated that *C*. *auris* colonies can undergo morphological transitions between pink, white, and dark purple colony phenotypes when grown on CHROMagar [19]. The switch frequencies observed for transitioning between these distinct *C*. *auris* phenotypes appear to be higher than the white-opaque switch frequencies observed for *C*. *albicans* [54,58]. In *C*. *albicans*, white-opaque switching is a heritable transition between 2 different cell types called “white” and “opaque” that have distinct virulence properties, mating competencies, and antifungal resistance properties [59]. White cells are round; are smaller than opaque cells; and form smooth, white, and shiny colonies on nutrient agar medium containing the red dye phloxine B; opaque cells, on the other hand, are elongated; are larger than white cells; and form pink, flat, and rough colonies on nutrient agar medium containing phloxine B [58]. It is unclear whether the phenotypic switch between pink, white, and dark purple colonies observed in *C*. *auris* is heritable. The initial report of this phenotypic switch in *C*. *auris* did not provide cellular images of the morphologies of the cells within the different colored colonies [19]. Based on the reported colony morphologies, we believe that it is possible that this phenotypic switch observed in *C*. *auris* is likely similar to the core phenotypic switch system observed in *C*. *glabrata* when it is grown on nutrient agar medium containing copper(II) sulfate or phloxine B [60]. In *C*. *glabrata*, 4 colony phenotypes were observed, namely the white, light brown, dark brown, and very dark brown phenotypes. The gradation of colors across colonies is believed to reflect the accumulation of copper sulfite, the by-product of copper(II) sulfate reduction. Similarly, the phenotypic switch between pink, white, and dark purple colonies observed in *C*. *auris* could also reflect distinct cellular oxidative/reductive states [19]. However, it is unknown whether this *C*. *auris* colony phenotypic switch is heritable and whether it is associated with virulence and/or antifungal resistance. Given the similarities observed between colony phenotypic switches of *C*. *auris* and *C*. *glabrata*, it seems likely that this switch in *C*. *auris* could be associated with the regulation of cellular redox states and adaptation to environmental stresses.

#### Filamentation

Filamentous (hyphal or pseudohyphal) cell growth of pathogenic *Candida* species is critical for fungal invasion of host tissues [55,56]. The transition between the yeast and filamentous growth forms of *C*. *albicans* has been intensively investigated. It was initially hypothesized that *C*. *auris* was unable to form true hyphae, but rather only developed pseudohyphae [32,44]. However, increasing evidence indicates that *C*. *auris* isolates can form true hyphae under specific circumstances.

Environmental factors including serum, N-acetyglucosamine (GlcNAc), and high levels of CO_2_ are potent inducers of filamentous growth in *C*. *albicans* [56]. It was recently determined that these factors did not induce filamentous growth in *C*. *auris* [41]. Thermotolerance and osmotolerance are distinguishing characteristics of *C*. *auris*. In 1 recent study, *C*. *auris* was grown on yeast extract peptone dextrose (YPD) medium supplemented with 10% NaCl. This condition induced the formation of elongated and pseudohyphal-like cells at both 37°C and 42°C [41]. Heat shock protein 90 (Hsp90) is an essential molecular chaperone that controls temperature-dependent filamentation in *C*. *albicans* [61]. Another study recently reported that treatment of *C*. *auris* cells with an Hsp90 inhibitor resulted in the formation of pseudohyphal-like cells [62]. Similar inhibition of Hsp90 in *C*. *albicans* resulted in filamentous growth, suggesting that certain regulatory mechanisms of filamentation are conserved, at least in part, between *C*. *albicans* and *C*. *auris*. These studies also indicate that certain *C*. *auris* isolates have the potential to undergo filamentation under specific environmental conditions.

**Fig 5:**
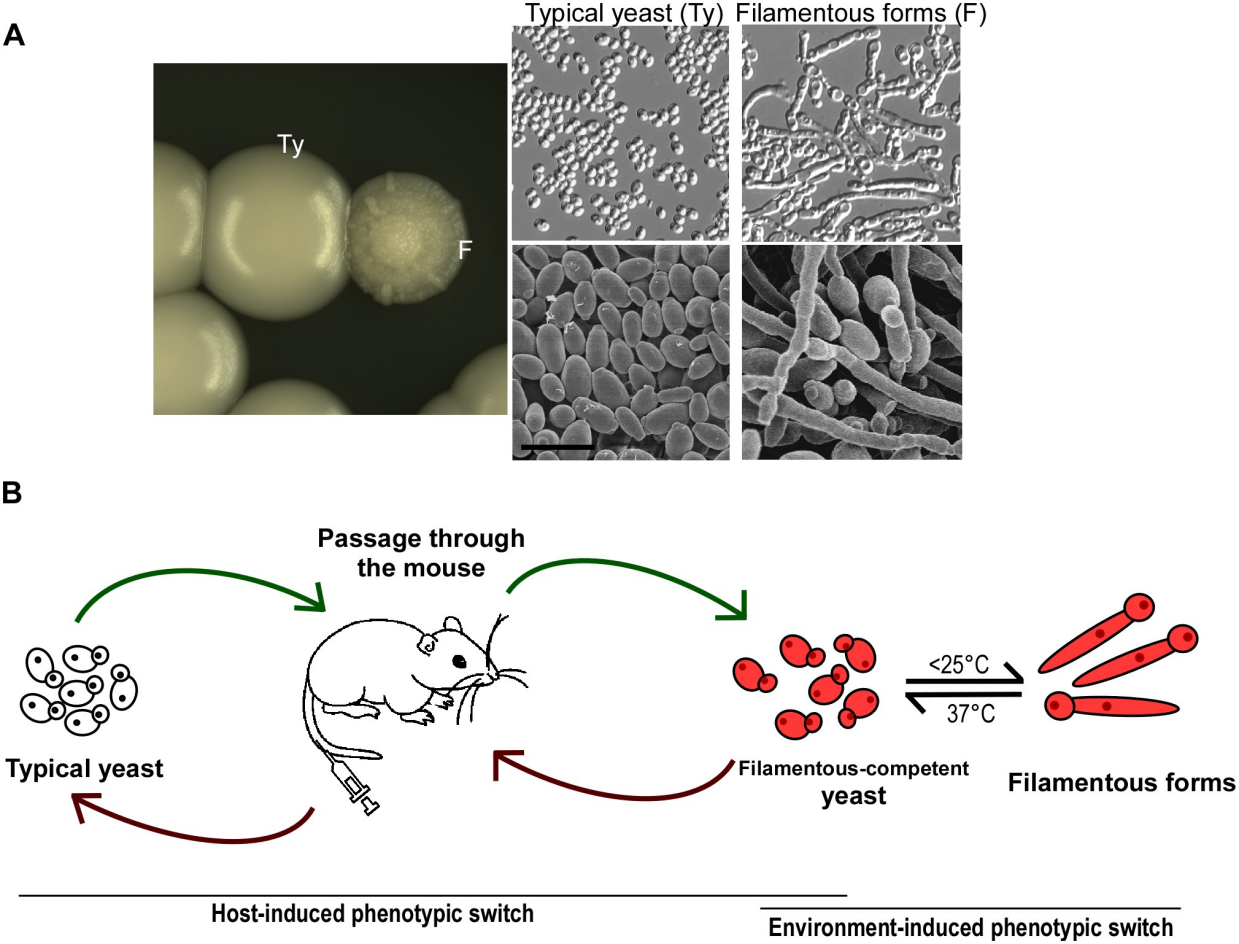
Morphological transitions in *C*. *auris*. (A) Colony and cellular morphologies of *C*. *auris* typical yeast form and filamentous-form phenotypes. Cells were grown on YPD medium. Images were adapted from [43]. (B) Known mechanisms for in vivo and in vitro phenotypic switching. Passage through the mouse mediates the switch between the typical yeast form and the filamentous competent yeast forms, whereas temperature mediates the in vitro switch between the filamentous competent yeast form and the filamentous forms. YPD, yeast extract peptone dextrose.

It was recently found that a subset of *C*. *auris* cells gained the ability to undergo filamentation after passage through the mouse in a systemic infection model ([43] and Fig 5). Three distinct cellular phenotypes were described in this study: typical yeast cells, filamentation-competent yeast cells, and filamentous-form cells. The typical yeast cells were locked in the yeast form and were unable to filament under in vitro culture conditions or upon filament-inducing environmental stimuli (e.g., medium and temperature changes or treatment with filamentation inducers for *C*. *albicans*). After passage through the mouse, a small proportion of typical yeast cells gained the ability to form filaments and were termed as “filamentation-competent yeast cells.” After recovering these yeast cells from mouse tissues and growing them on YPD or Lee’s medium at temperatures of 25°C or lower, these *C*. *auris* cells underwent robust filamentation and were termed as “filamentous-form cells.” Surprisingly, microscopy analysis indicated that these filamentous-form *C*. *auris* cells appeared morphologically similar to true hyphae formed by *C*. *albicans* [43]. Interestingly, of the conditions tested, the low temperature condition (<25°C) was most conducive for filamentous growth, while the human physiological temperature (37°C) repressed filamentous growth in *C*. *auris*. This phenomenon is in contrast to that observed in *C*. *albicans* where cells predominantly grow in the yeast form at low temperatures and the filamentous form at human physiological temperatures [56]. These findings suggest that filamentous morphologies of *C*. *auris* could exist in the environment and on the host skin surface where the temperature is lower than inside the host.

Switching between the typical yeast form and the filamentation-competent yeast form of *C*. *auris* was a rare event, but when it did occur, it was heritable [43]. Switching between the filamentation-competent yeast cells and filamentous-form cells, on the other hand, was nonheritable and dependent on the environment [43]. These findings indicate that once *C*. *auris* cells obtain the ability to filament, they can develop robust filamentous cells upon environmental stimuli (e.g., growth at low temperatures). This heritable switch between the typical yeast form cells and filamentation-competent yeast form cells is akin to the white-opaque phenotypic switching system in *C*. *albicans* [63]. Similar to the filamentation-competent yeast form cells of *C*. *auris*, both white and opaque cells of *C*. *albicans* can maintain their cell identities for many generations. It remains to be investigated whether the mechanisms of this cellular memory in *C*. *auris* are genetically or epigenetically regulated. Nonetheless, these 3 *C*. *auris* cell types appear to form a 3-way phenotypic switching system that consists of a heritable transition between the typical yeast cells and the filamentation-competent yeast cells and a nonheritable transition between the filamentation-competent yeast cells and the filamentous-form cells (Fig 5B).

The yeast and filamentous cells of *C*. *auris* differ in a number of biological aspects including global gene expression profiles, expression of virulence factors, and virulence in a mouse infection model [43]. Interestingly, a large set of metabolism-related genes was differentially expressed between *C*. *auris* yeast and filamentous cells. Genes involved in sugar transportation, glycolysis, and the Krebs cycle were up-regulated in filamentous cells, suggesting that general metabolic processes are more active in filamentous cells relative to yeast cells of *C*. *auris*.

Based on yeast carbon base–BSA (YCB–BSA) assays that detect secreted aspartyl protease (Sap) activity, *C*. *auris* typical yeast cells and filamentation-competent yeast cells displayed higher levels of Sap production relative to filamentous-form cells when grown at 25°C [43]. All 3 cell types exhibited similar levels of Sap secretion at 37°C, likely due to the fact that filamentous-form cells converted “en masse” to filamentation-competent yeast cells at this temperature [43]. This notable difference in Sap secretion may influence the abilities of the typical yeast and filamentous-form cells to adapt to diverse ecological niches. Given that filamentous-form cells compared to typical yeast form cells of *C*. *auris* produce lower levels of Saps, which are important virulence factors, the filamentous form may be better adapted to colonizing the skin of the host as a commensal. Consistent with this idea, it has been suggested that *C*. *auris*, unlike *C*. *albicans*, is a primary colonizer of the skin rather than the GI tract of humans [10].

Why has *C*. *auris* evolved a filamentation-competent yeast form? This is an important unanswered question that should be explored in future studies. One possible explanation is that the 3-way phenotypic switching system in *C*. *auris* consisting of the typical yeast, filamentation-competent yeast, and filamentous forms is much more complex and versatile than the 2-way switching systems observed in *C*. *albicans*, such as the yeast filament and the white-opaque transitions. Compared to *C*. *albicans*, this added phenotypic plasticity could allow *C*. *auris* to more efficiently adapt to the ever-changing environment.

#### Biofilm development

Biofilms are structured microbial communities that form on abiotic and biotic surfaces and are embedded in an extracellular matrix [64]. The biofilm mode of growth is the preferred state for microorganisms in natural ecological niches. In a clinical setting, a biofilm formed on human tissue (e.g., on a mucosal layer) or on an implanted medical device (e.g., a central venous catheter) can serve as a source of infection that can spread to other parts of the body [65]. It has been found that *C*. *auris* can develop biofilms on surfaces, although its biofilms are relatively weak compared to those formed by *C*. *albicans* [12]. *C*. *auris* biofilm cells, similar to *C*. *albicans* biofilm cells, however, have been shown to exhibit high levels of resistance to antifungal agents compared to their free-floating (planktonic) cell counterparts.

Biofilm formation abilities vary across *C*. *auris* isolates and clades [44]. Although both the aggregated and non-aggregated *C*. *auris* cell types are able to develop biofilms, the latter have been shown to form more robust biofilms [44]. Interestingly, time course RNA-sequencing experiments identified genes encoding putative adhesins, efflux pumps, and virulence factors to be up-regulated during *C*. *auris* biofilm development [66]. Although the roles of *C*. *auris* biofilms are less understood than those of biofilms formed by other *Candida* species, *C*. *auris* biofilms certainly contribute to the virulence, antifungal resistance, and survival properties of *C*. *auris* in the environment and likely in the host. Therefore, the development of therapeutic approaches to target *C*. *auris* biofilms both in patients and in the environment is an important area for future research.

### Antifungal resistance mechanisms

One important reason that *C*. *auris* is considered to be a “superbug” and is increasingly becoming a threat to human health is its intrinsic resistance to 1 or more classes of antifungal drugs available in the clinic [22,35]. Based on the conservative antifungal drug break points for *C*. *albicans* and other *Candida* species, most isolates of *C*. *auris* are resistant to fluconazole. A subset of *C*. *auris* isolates has high minimum inhibitory concentrations (MICs) than that of amphotericin B and echinocandin compounds, and some *C*. *auris* strains are resistant to all available classes of antifungal drugs [10,11]. A comparative study of European Committee on Antimicrobial Susceptibility Testing (EUCAST) and Clinical and Laboratory Standards Institute (CLSI) methods revealed that *C*. *auris* isolates have a remarkably similar fluconazole resistance but a wide range of MICs for the other antifungal drug classes [67]. It is noteworthy that the closely related species to *C*. *auris*, *C*. *haemulonii*, and *C*. *lusitaniae* are also often resistant to 1 or multiple antifungal drug classes [11,68]. This observation suggests that *C*. *auris*, C. *haemulonii*, and *C*. *lusitaniae* have similar genetic mechanisms for their antifungal resistance properties; *C*. *haemulonii* and *C*. *lusitaniae*, however, are infrequently isolated as infectious agents.

Ergosterol is the major sterol component of fungal membranes and is the target of the azoles (e.g., fluconazole) and the polyenes (e.g., amphotericin B) [69,70]. The first-line antifungal drug in the clinic, fluconazole, inhibits cellular ergosterol biosynthesis by targeting the fungal cytochrome P450-dependent enzyme lanosterol demethylase that is essential for the production of ergosterol. *ERG11* encodes lanosterol demethylase in the *Candida* species. Interestingly, 3 hot spot mutations (Y132F, K143R, and F126L or VF125AL) have been found in Erg11 in fluconazole resistant *C*. *auris* strains of different genetic clades [10].

Although isolates of *C*. *auris* that are resistant to fluconazole and amphotericin B are common, echinocandin-resistant isolates (e.g., caspofungin) are relatively rare [71]. *FKS1* encodes the catalytic subunit of 1,3-beta-D-glucan synthase that is critical for cell wall synthesis and maintenance in *Candida* species [72,73]. Isolates of *C*. *auris* with an S639F mutation in Fks1 were caspofungin resistant, while other isolates harboring a wild-type Fks1 were susceptible to caspofungin at human therapeutic doses [74].

### Virulence and animal models

Infections by *C*. *auris* can occur at multiple body sites, including the skin, urogenital tract, and respiratory tract of humans. *C*. *auris* infections can disseminate to the bloodstream, and when this occurs, they are associated with high mortality rates [7,16,23,35,39]. Recent reports have demonstrated that *C*. *auris*, similar to *C*. *albicans*, expresses several known virulence factors, including Saps and lipases to degrade and invade host tissues [41,43]. Comparative studies in animal models indicate that *C*. *auris* is less virulent than *C*. *albicans*, both in the murine disseminated infection model and in the invertebrate *G*. *mellonella* infection model [32,41]. However, *C*. *auris* is significantly more virulent than *C*. *glabrata* and *C*. *haemulonii* in the murine infection model [75,76]. This decrease in virulence relative to *C*. *albicans* is likely due to the fact that *C*. *auris*, along with *C*. *glabrata* and *C*. *haemulonii*, is unable to develop hyphae or pseudohyphae in the mammalian host that play critical roles in tissue invasion during infections [43]. Another possible reason for the relatively low virulence of these 3 species compared to *C*. *albicans* is that they are all haploid microorganisms, while natural *C*. *albicans* isolates are diploid. Consistent with this idea, a fluconazole-induced haploid *C*. *albicans* strain was found to be much less virulent than its diploid counterpart [77]. In 1 recent study, all cell types of *C*. *auris* that were initially injected into a mouse (regardless of whether they changed into another cell type) exhibited similar levels of virulence [43]. We propose that filamentous cells of *C*. *auris* are unlikely to contribute to virulence in systemic infections but are more likely to play roles in colonizing the skin and environmental surfaces. This idea is supported by a study that found that *C*. *auris* filamentous cells produce fewer virulence factors (e.g., Saps) compared to *C*. *auris* yeast-form cells [43].

Some isolates of *C*. *auris* can form aggregates under both in vitro and in vivo conditions. Cell aggregation could benefit *C*. *auris* by allowing fungal cells to evade the host immune system, persist in host tissues, and have increased levels of antifungal tolerance [75]. Unlike *C*. *albicans* cells, neutrophils are poorly recruited to *C*. *auris* cells, are not effective at killing *C*. *auris* cells, and do not form neutrophil extracellular traps (NETs) [78]. Evasion of the host neutrophil attack seems to be an important *C*. *auris* survival strategy within the host. The ability of *C*. *auris* cells to form aggregates seems likely to hinder the host innate immune response by creating a protective physical barrier for *C*. *auris* cells from the environment.

Given that *C*. *auris* is such an important and emerging pathogen in clinical settings, effective animal models are needed to investigate its pathogenesis and biology. Other than the murine model and the *G*. *mellonella* model, several other animal models, including *Drosophila melanogaster* and *Caenorhabditis elegans* invertebrate models, have been used to evaluate *C*. *auris* pathogenesis and the effects of antifungal therapies on treating *C*. *auris* infections [79,80]. A study on the use of Toll-deficient flies to model *C*. *auris* and *C*. *albicans* infections found that *C*. *auris* infections had significantly higher mortality rates than those of *C*. *albicans* [79]. Using a *C*. *elegans* model, another study found that sulfamethoxazole and itraconazole synergistic treatment was effective against *C*. *auris* infections caused by specific multidrug-resistant *C*. *auris* strains [80]. Overall, invertebrate animal infection models are useful in providing a fast and inexpensive means to study pathogenesis and antifungal resistance in *C*. *auris*.

### Genomics and genetics

*C*. *auris* is a haploid fungus. As of April 2020, the genomic sequences of over 700 *C*. *auris* isolates are available on the National Center for Biotechnology Information (NCBI) genome database (https://www.ncbi.nlm.nih.gov/genome/) [81]. The genome of *C*. *auris* B8441 was sequenced by the CDC [10], and its sequence information and annotation is available on the Candida Genome Database (CGD) database (https://www.candidagenome.org). *C*. *auris* has 7 chromosomes, and the genome sizes of isolates range from 12.1 Mb to 12.7 Mb [13]. Based on genomic and RNA-sequencing information, it is estimated that *C*. *auris* has approximately 5,500 predicted genes [13,14].

Although sexual reproduction has not been observed in *C*. *auris* to date, the mating type loci (*MTL***a** and *MTL*α) and most mating and meiosis genes are found in the *C*. *auris* genome [13]. These loci are generally well conserved and share high structural and sequence similarities within the CTG clade species [13]. The *MTL* loci of *C*. *auris*, similar to *MTL* loci of other CTG clade species, contain several “non-sexual” genes such as the phosphatidylinositol kinase encoding gene *PIK1*, the oxysterol binding protein encoding gene *OBP1*, and the poly(A) polymerase encoding gene *PAP1*. All *C*. *auris* strains isolated to date have contained either the *MTL***a** or the *MTL*α locus. Isolates of clades I and IV have an “a” mating type (*MTL***a**), whereas isolates of clades II and III have an “α” mating type (*MTL*α) [13]. Given that both MTL are present in *C*. *auris*, it seems likely that *C*. *auris* should be able to mate, but that we have simply not yet identified a mating conducive niche for this fungus.

Comparative genomic analyses indicate that the *C*. *auris* genome contains conserved genes within the CTG clade that are associated with virulence and antifungal resistance [13,14]. For example, genes encoding the Saps, components of the ergosterol biosynthesis pathway, the MFS transporter Mdr1, and the transcriptional regulators Upc2 and Tac1 (including Tac1A and Tac1B) are all present in the *C*. *auris* genome. Intriguingly, a recent study found that mutations in *TAC1B* are associated with increased fluconazole resistance in *C*. *auris* [82]. In addition, there are only a small number of unique genes in *C*. *auris* that are absent in its closely related CTG clade species. These *C*. *auris*-specific genes include genes encoding oligopeptide and ATP-binding cassette (ABC) transporters, further contributing to its intrinsic antifungal-resistant nature [13].

### Origins

Four major clades of *C*. *auris* (I, II, III, and IV initially isolated from South Asia, East Asia, South America, and South Africa, respectively) and a potential fifth clade isolated from Iran have been described to date [10,34]. Whole-genome sequencing analysis revealed that the first 4 genetically distinct clades of *C*. *auris* emerged independently and nearly simultaneously at different locations across 3 continents [10]. Although *C*. *auris* is closely related to other pathogenic *Candida* species that can be found in the environment, *C*. *auris* has not been found to exist in natural environmental settings. One study from the Netherlands reported isolating *C*. *auris* from swimming pools [83], but in these cases, the fungus likely originated from pool visitors. Two recently published perspective articles have comprehensively discussed hypothetical ecological origins of *C*. *auris* [21,47]. It has been proposed that the emergence of *C*. *auris* may have resulted from climate change, specifically global warming [21]. The authors propose that *C*. *auris* was an environmental fungus before it evolved to be a human pathogen as the climate increased in temperature. Since *C*. *auris* is highly tolerant of high ambient temperatures and hypersaline conditions, the authors suggest that wetlands could be the natural ecological niche of *C*. *auris* before it became associated with warm-blooded animals and humans. Moreover, the increased use of antifungal agents in medicine and agriculture likely further contributed to the emergence of *C*. *auris* as well as other antifungal-resistant and antifungal-tolerant fungal species [21].

Over the past several decades, more and more fungal pathogens have been emerging to threaten humans, animals, and plants. Human activities, including those that result in global warming, may be supporting environments that allow for the evolution of fungal characteristics that are conducive to host colonization and infection. It seems likely that novel fungal pathogens will similarly emerge in the future due to changes in global temperatures, atmospheric CO_2_ levels, humidity, and other alterations to natural environmental niches [84].

## Conclusion and open questions

*C*. *auris* is a new public global health threat. Despite the close phylogenetic relationship of *C*. *auris* to other pathogenic *Candida* species, *C*. *auris* has many unique characteristics in its biology, genetics, epidemiology, antifungal resistance, virulence, host adaptation, and transmission.

While *C*. *auris* has garnered significant scientific attention recently, there are many unanswered questions related to its emergence and biology. What are the original environmental reservoirs for *C*. *auris*? How did isolates with different genetic backgrounds emerge nearly simultaneously worldwide? How did multidrug resistance evolve in *C*. *auris*? What enables *C*. *auris* to persist in clinical settings for long periods of time? Is *C*. *auris* capable of sexual or parasexual reproduction and, if so, did this contribute to its emergence as a pathogen? Significant research efforts are needed to begin to answer these questions. We need to explore the basic biology and genetic bases of antifungal resistance and pathogenicity in *C*. *auris*. As we gain mechanistic knowledge on *C*. *auris*, we should be able to develop rapid and accurate detection methods to distinguish *C*. *auris* from other *Candida* species, which will help with diagnosing *C*. *auris* infections. We will also be able to develop new disinfection protocols for the effective removal of *C*. *auris* from surfaces, which will prevent future outbreaks. Finally, we need to develop novel, safe, and effective antifungals and treatment strategies with diverse drug targets to combat infections cause by *C*. *auris* as well as other existing and soon-to-be emerging fungal pathogens.
